# Transcriptome analysis suggests the cell wall as the primary target of the antilisterial effect of carrots

**DOI:** 10.1128/spectrum.02091-25

**Published:** 2025-11-25

**Authors:** Jana Helen Walter, Marc J. A. Stevens, Sophie Franzmeier, Thomas Nothnagel, Frank Dunemann, Claudia Guldimann, Irmak Şah

**Affiliations:** 1 Chair for Food Safety and Analytics, Faculty of Veterinary Medicine, Ludwig-Maximilians-University (LMU) Munich, Competence Center for Food Safety 84532 , Munich, Germany; 2 Institute for Food Safety and Hygiene, Vetsuisse Faculty, University of Zurich 30843, Zürich, Switzerland; 3 Section of Clinical & Comparative Neuropathology, Institute of Veterinary Pathology, Center for Clinical Veterinary Medicine, Ludwig-Maximilians-Universität München 208059, Munich, Germany; 4 Julius Kühn-Institut (JKI), Federal Research Centre for Cultivated Plants, Institute for Breeding Research on Horticultural Crops 98882, Quedlinburg, Germany; The Pennsylvania State University, University Park, Pennsylvania, USA

**Keywords:** *Listeria monocytogenes*, antilisterial effect, carrots, cell wall integrity, transcriptome

## Abstract

*Listeria monocytogenes* is a serious foodborne pathogen. To prevent *L. monocytogenes* contamination, comprehensive hygiene protocols are implemented during food processing. Moreover, targeted antimicrobial interventions are desirable. Carrots have long been recognized for their antimicrobial effects against *L. monocytogenes*. However, the underlying mechanisms behind this effect have not been elucidated so far. In this study, we aimed to (i) identify the carrot accessions exhibiting the strongest antimicrobial effects from a selection of 52 different accessions and (ii) characterize the transcriptomic response of three *L. monocytogenes* strains to stress induced by carrot juice. After screening 52 different carrot accessions on *L. monocytogenes*, of which 19 killed 4-log_10_ of *L. monocytogenes* within 3 min, we conducted a transcriptomic study using three highly effective carrot accessions to analyze gene expression changes in response to carrot juice exposure. Exposure to carrot juice caused significant changes in the expression of genes associated with the structure and integrity of the cell wall, two-component regulatory systems, and ABC transporters, highlighting the complex response of *L. monocytogenes* to carrot juice. Furthermore, we observed that several genes associated with nisin resistance were significantly differentially expressed in response to carrot juice stress.

**IMPORTANCE:**

*Listeria monocytogenes* is a major concern for food safety due to its remarkable resilience to a wide range of stress conditions in food matrices and food-processing environments. Given that carrots have previously been shown to exert antimicrobial effects against *L. monocytogenes*, we sought to further investigate this phenomenon. Our results demonstrate that carrot juice triggers differential expression of genes involved, among others, in maintaining cell wall structure and integrity. These findings suggest that carrots may represent a promising natural intervention strategy to mitigate *L. monocytogenes* contamination, aligning with the growing consumer demand for natural food additives.

## INTRODUCTION

*Listeria monocytogenes* is one of the most important foodborne pathogens worldwide, causing 2,952 cases of clinical listeriosis, including 335 deaths in Europe in 2023 (1). It is the fifth most reported zoonosis in humans in the European Union (1) and poses a significant public health concern due to its high case fatality rate.

Common examples of foods affiliated with *L. monocytogenes* contamination are products of meat origin, like ready-to-eat deli meat, products of meat origin other than fermented sausage and fish (1). Recurring outbreaks (2–4) underscore the serious impact of *L. monocytogenes* on public health and the need for additional food-safety strategies. *L. monocytogenes* easily adapts to various stress conditions commonly encountered in food production and processing environments (5), including high salt concentrations, low temperatures, low water activity (aw), and a low pH (6). This resilience makes *L. monocytogenes* particularly difficult to control in the food production and processing industry and within the food matrix, particularly when foods do not undergo a heat inactivation step prior to consumption.

Naturally occurring antimicrobials have been used to control *L. monocytogenes* in the food industry, for example, nisin (7), and essential oils with antilisterial properties (8, 9). However, the usage of essential oils might alter the flavor of the product (10), whereas nisin might be degraded by proteases or accumulate in fatty phases (11). Beyond this, *L. monocytogenes* has the ability to adapt to not only the currently used antimicrobial agents (12, 13) but also to other measures of prevention, for example, the use of disinfectants (14). Therefore, it becomes imperative to explore comprehensive measures aimed at mitigating the contamination risk to further enhance food safety. Consumer demands for natural and minimally processed foods are driving the need for natural antimicrobial agents as alternatives to synthetic preservatives. Therefore, many naturally occurring antimicrobial agents of different origins are being explored and tested for their efficacy against bacteria *in vitro* and in the food matrix (15). Some examples include lignan-containing maple products that inhibit the biofilm formation of *L. monocytogenes* (16), the potential antimicrobial effect of the marine macroalga *Ericaria selaginoides* against *L. monocytogenes* in a fresh-cheese matrix (17), the antilisterial effect of grape-seed extract on apple, celery, and cantaloupe (18), among others (19–21).

Carrots have long been recognized for their antibacterial effects against *L. monocytogenes* (22, 23). Since the discovery of this effect, different approaches have been used to try to identify both the mechanism of action and the active agent (24). Carrots contain varying concentrations of several candidate antimicrobial substances (25), such as polyacetylenes (26), laserines (27), or anthocyanins (28). To the best of our knowledge, currently, none of these substances is the sole causative agent responsible for the observed antilisterial effect. It has been observed that the effect is heat sensitive and shows enhanced efficacy at lower temperatures (29). Another recent study found that UV-C treatment increased the antilisterial effect of carrots (30), and morphological changes in *L. monocytogenes* were observed under stress conditions caused by carrot juice (31). Furthermore, fluorescence microscopy findings indicated that the effect was bactericidal (30). Although the effect has yet to be fully elucidated, the influence of the carrot variety and the molecular stress-response mechanisms used by *L. monocytogenes* remains unclear.

In this study, we investigated the antimicrobial effect of different carrot accessions against three strains of *L. monocytogenes*. We used a diverse set of carrot accessions, including modern varieties, open-pollinated varieties, landraces, breeding lines, and wild relatives. These originated from gene banks, breeders, or the breeding program at the JKI Institute. An “accession” refers to a distinct, documented sample preserved within a gene bank or collection. As the majority of the carrot materials used in this study originate from gene banks and the JKI collection, the most scientifically appropriate collective term to describe them is “accessions.” We also aimed to elucidate the molecular mechanism underlying this interaction. By examining the gene expression profiles of three *L. monocytogenes* strains in response to carrot juice treatment, we identified specific genes and pathways that are a part of the stress response mechanisms and contribute to adaptation under carrot-induced stress. This approach provides valuable insights into the regulatory pathways that are involved in the bacterial stress response to carrot-based antilisterial stress. Interventions in the expression of these mechanisms could potentially inform new targets to improve food safety.

## RESULTS

### Nineteen of 52 carrot accessions significantly reduced *L. monocytogenes* LL195 counts

In a first step, we analyzed the antilisterial activity of 52 carrot accessions. Nineteen accessions reduced the 4-log_10_ *L. monocytogenes* LL195 to below the detection limit within 3 min, eight accessions achieved this within 15 min, six within 30 min, and seven within 120 min of incubation. Three additional accessions showed a reduction, but the bacteria were not completely eradicated, even after 120 min of incubation (Table 1). Notably, only three carrot accessions showed no detectable reduction in bacterial counts. The remaining six accessions, five of them wild relatives, were excluded from further analysis since juice extraction was not possible for those accessions.

**TABLE 1:** Results of screening experiments performed with *L. monocytogenes* LL195 on carrots from three different harvesting periods^*a*^

		Harvest 2021				Harvest 2022			Harvest 2023		
		Inoculation level of 4-log_10_ CFU/mL				Inoculation level of 7-log_10_ CFU/mL			Inoculation level of 7-log_10_ CFU/mL		
Number	Name	3 min	15 min	30 min	120 min	3 min	15 min	30 min	3 min	15 min	30 min
1	Afghan Purple	+++	+++	+++	+++	N/A^*b*^					
2	Anthonina	+++	+++	+++	+++	–	–	–			
3	Berlicumer Bercoro	–	–	–	–	–	–	–	–	–	–
4	Berlikum Perfecta	++	+++	+++	+++						
5	BetaIII	–	+	++	+++						
6	Bitolski	–	+	++	+++						
7	Blanche 1/2 longue des vosges	+	+++	+++	+++						
8	Brasilia	+++	+++	+++	+++	–	+/–	++			
9	D. c. maximus	–	++	+++	+++						
10	D. c. carota	N/A^*c*^									
11	D.c. gummifer subsp. gummifer	N/A^*c*^									
12	D.c. gummifer subsp. commutatus	N/A^*c*^									
13	D. c. carota	+++	+++	+++	+++	–	+++	+++	+	+++	+++
14	D. c. carota	+++	+++	+++	+++	–	–	–			
15	D. c. gummifer	+++	+++	+++	+++	–	–	–			
16	D. c. carota	N/A^*c*^									
17	D. c. azoricus	+	+	+++	+++	–	–	–			
18	Deep Purple	+	+	++	+++						
19	Gajar	–	+	++	+++						
20	Hakata Kintoki	N/A^*c*^				–	–	–			
21	Himuro Fuyugosi Gosun No.2	+	+++	+++	+++						
22	Kampino	++	+++	+++	+++						
23	Kuettiger	+++	+++	+++	+++	–	–	–			
24	Long Red	+++	+++	+++	+++						
25	Nantes Apollo	–	–	–	++						
26	Nantes Liva	++	+++	+++	+++						
27	Nantes Palisade	–	++	+++	+++						
28	Nutrired	+++	+++	+++	+++	–	–	–			
29	JKI-BL 12/87	+++	+++	+++	+++	–	–	–			
30	Pariser Markt	–	–	–	–	–	–	–	–	–	–
31	Presto	–	–	–	–	–	–	–	–	–	–
32	Rotin	+	+++	+++	+++						
33	Santa Cruz	-	+	++	+++						
34	Sapporo Futo	+++	+++	+++	+++	–	–	–			
35	Senta	+++	+++	+++	+++	–	–	–			
36	Short n'Sweet	++	+++	+++	+++						
37	Stratova	+++	+++	+++	+++	–	–	–			
38	Viking	+++	+++	+++	+++	–	–	–			
39	Vita Longa	–	+	++	+++						
40	Western Red	++	+++	+++	+++						
41	White Satin	–	–	–	+						
42	Winterperfection	–	+	+++	+++						
43	Yellowstone	+++	+++	+++	+++	–	–	–			
44	GAT 52121	+++	+++	+++	+++	++/–	+++	+++	+++	+++	+++
45	D. c. carota HRIGRU 007301	N/A^*c*^				–	–	–			
46	D. c. carota GRCGGB 11076	+	++	+++	+++						
47	AZ 226/10	+++	+++	+++	+++	–	+/++	+++	+++	+++	+++
48	AZ 227/10	+++	+++	+++	+++	++	+++	+++			
49	AZ 75/07	+	+	+++	+++	N/A^*b*^					
50	AZ-Tam-13	+	++	++	+++						
51	Vitaminaja	–	–	–	+						
52	Yamanouchi Ishyaku Senko	+++	+++	+++	+++	–	–	–			

^*a*^ (–) no observable antilisterial effect, (++) approximately 2-log reduction, (+) approximately 3-log reduction, (+++) below the detection limit.

^*b*^ N/A, There were not enough seeds to plant again.

^*c*^ N/A, This could not screen due to spoilage/could not juice.

In the first year, the full set of carrot accessions was screened. To determine the most effective carrot accessions and to test whether the effect was stable over several seasons, a second screening from another harvest was performed using the 19 carrot accessions that showed the strongest antilisterial effect. In this second screening, a higher inoculation rate of 7-log_10_CFU/mL was chosen to identify the carrots with the strongest effect. Eleven of the 19 carrot accessions exhibited no observable antilisterial effect of the 7-log_10_ CFU/mL in this screening.

Although no carrot accession showed an effect within the first 3 min of exposure, three accessions reduced *L. monocytogenes* LL195 below the limit of detection within 15 min, and one accession achieved this within 30 min. The three carrot cultivars, 3, 30, and 31, that had no antilisterial effect on *L. monocytogenes* LL195 in the first screening remained ineffective over the harvesting periods. Overall, three carrots showed a stable, strong effect across 3 consecutive years.

We selected three carrot accessions, 13, 44, and 47, which exhibited the strongest antilisterial effect, and three carrot accessions, 3, 30, and 31, without an observable antilisterial effect for transcriptomic analyses. Of the three accessions with high effects, no. 13 is a wild relative from Bretagne, France; no. 44 is a landrace from Turkey; and no. 47 is a breeding line from an anthocyanin carrot program with Turkish-Syrian origin. The latter are three commercial carrot cultivars: No. 31 (Presto, modern), no. 3 (Berlikumer, old cultivar), and no. 30 (Paris Market, old cultivar).

**Fig 1:**
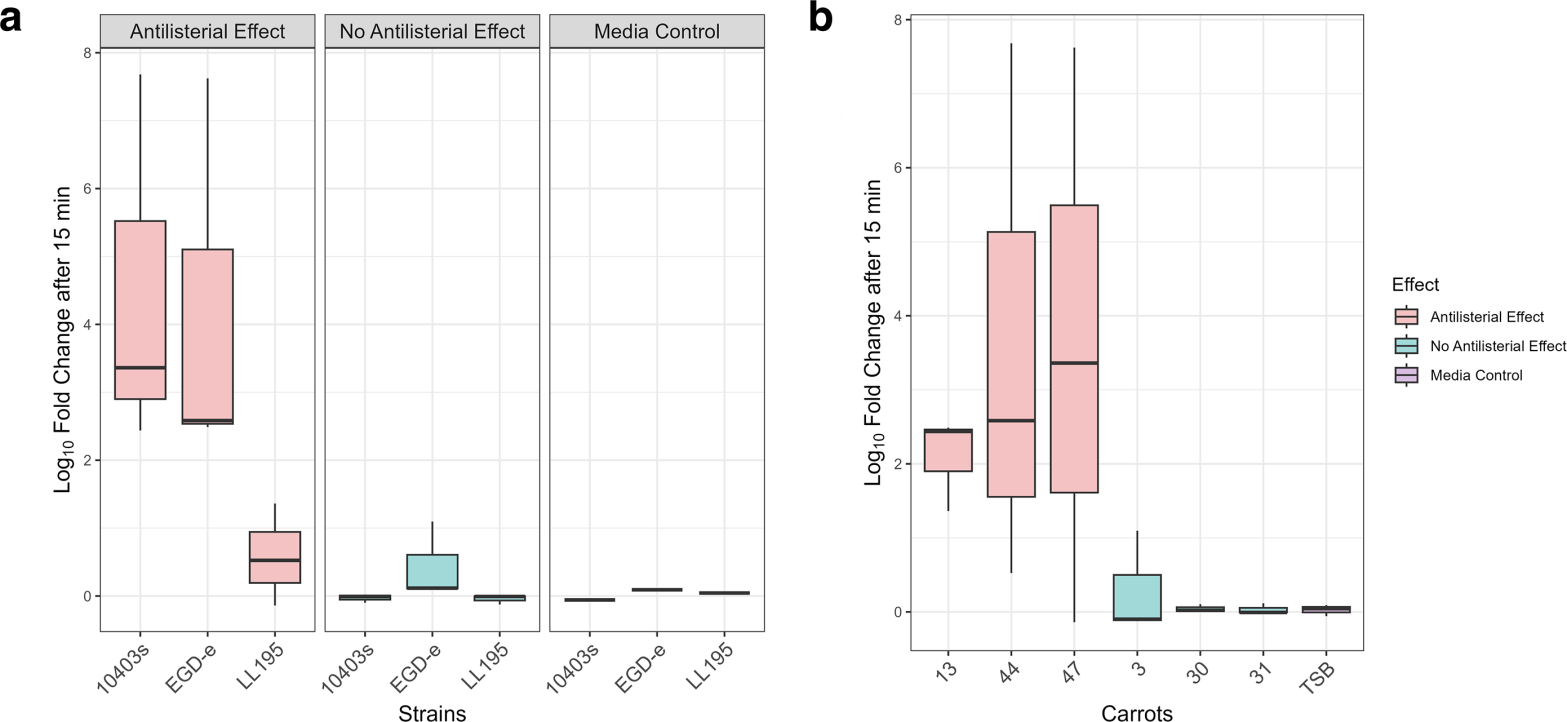
The log_10_ fold change of three strains of *L. monocytogenes*, after 15 min of incubation in 1:25 diluted carrot juice. (**a**) The figure shows the results sorted by strain. (**b**) The figure shows the results sorted by individual carrots. The box plots represent the median (line), the 25th and 75th percentiles (box), and the minimum and maximum values (whiskers).

In a third harvest, only these six accessions were cultivated and tested. No. 44 and 47 eradicated all *L. monocytogenes* within 3 min, whereas no. 13 killed all *L. monocytogenes* within 15 min after incubation. The three ineffective carrots remained so (Fig. 1B).

Although the screenings were performed in pure carrot juice, a 1:25 dilution of carrot juice was used for the RNA-seq experiment to ensure significant stress but avoid complete eradication of the cells. Even with the 1:25 dilution of carrot juice, high eradication rates of all three strains of *L. monocytogenes* were achieved (Fig. 1)

Additionally, freezing was attempted to preserve the carrot juice for a longer period; however, frozen carrot juice gradually lost its efficacy (data not shown). Further research is needed to investigate this loss of efficacy over time, both for frozen and unfrozen juice.

### Differentially expressed genes in *L. monocytogenes* strains exposed to the carrot-induced stress

**Fig 2:**
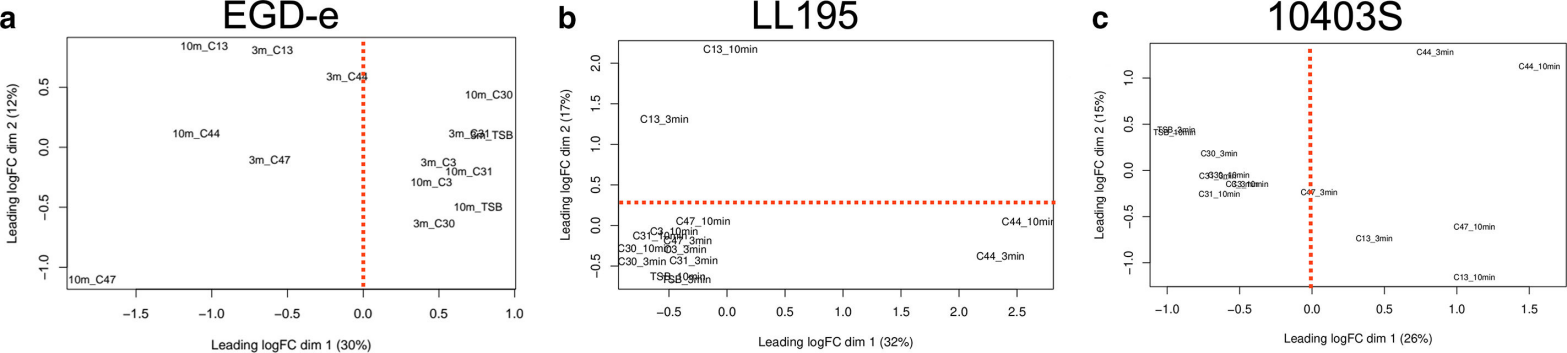
Multi-dimensional scaling of the RNA-seq results. Three strains of *L. monocytogenes* were exposed to carrot juice for 3 and 10 min. The effect of the three selected carrot accessions with (no. 13, 44, and 47) and without effect (no. 3, 30, and 31) and the negative control (TSB) on the cumulative transcriptome of the three *L. monocytogenes* strains used is shown. (**a**) The figure shows the results for *L. monocytogenes* EGD-e, (**b**) The figure shows results for LL195, and (**c**) shows the results for 10403S.

Three strains of *L. monocytogenes* were individually exposed to juice from three carrots with and three carrots without effect. The carrot juice was diluted 1:25 to achieve a level of stress that causes approximately 1-log_10_ CFU/mL reductions from the carrots with an effect. Samples for transcriptome analyses were taken after 3 and 10 min to detect both the immediate response and early adaptive mechanisms. Multi-dimensional scaling of the transcriptome profile showed a clustering of samples derived from cultures after exposure to juice from the antilisterial carrots no. 13, 44, and 47, and of samples from cultures treated with juice from carrots without effect, that is, no. 3, 30, 31, and the negative control in TSB (Fig. 2). A closer look revealed differences specific to carrots and bacterial strains. Carrot no. 13, as well as carrot no. 44, had a pronounced effect on *L. monocytogenes* LL195, whereas carrot no. 47 had a stronger effect on *L. monocytogenes* EGD-e (Fig. 2). Notably, *L. monocytogenes* LL195 was more resistant to carrot juice than the two other strains, EGD-e and 10403S.

All significantly differentially expressed genes are listed in Tables S1 to S3. For a more complete view of the global transcriptomic changes in the three strains, a comparative analysis was conducted by merging RNA-seq data obtained from both time points. In *L. monocytogenes* EGD-e, 161 genes were up- and 71 genes were down-regulated after exposure to carrot juice with an effect. In 10403S, 115 genes were up- and 26 genes were down-regulated, and in LL195, 85 genes were up- and 11 genes were down-regulated (Table 2). An excerpt of all significantly differentially expressed genes is presented in Table 3. Genes were included in Table 3 based on the following criteria: a false discovery rate (FDR) < 0.05 and a log_2_ fold change (log_2_FC) > |1|, plus one of the following: (i) significant representation in KEGG analysis, (ii) occurrence of several genes within the same operon, or (iii) known or hypothetical function in environmental stress response. Genes annotated as hypothetical with no further information were excluded.

**TABLE 2:** Summary of transcriptomic analysis showing the number of upregulated and downregulated genes at each time point for each strain^*a*^

Strain	Expression	Time point (min)	Number of differentially expressed genes	Number of differentially expressed genes unique to this time point
10403S	Up	3	52	8
		10	119	75
10403S	Down	3	1	0
		10	33	32
EGD-e	Up	3	87	19
		10	161	93
EGD-e	Down	3	16	7
		10	86	77
LL195	Up	3	18	2
		10	43	27
LL195	Down	3	0	0
		10	30	30

^*a*^ The figure also highlights genes uniquely expressed at specific time points within each strain of *L. monocytogenes*.

**TABLE 3:** Significantly differentially expressed genes of *L. monocytogenes* across all three strains and both time points (for the full data set, refer to Tables S1 to S3)

ORF	Function	Strain	Time point	Log_2_FC	FDR
*L. monocytogenes* LL195					
BN389_27200	Multidrug resistance ABC transporter ATP-binding/permease protein BmrA	LL195	10	4,19	3,00E-3
BN389_27200	Multidrug resistance ABC transporter ATP-binding/permease protein BmrA	LL195	3	3.36	2.00E-02
BN389_06440	Uncharacterized ABC transporter ATP-binding protein Mb1304c	LL195	combined	1.56	2.00E-03
BN389_08280	ywaC (*P*)ppGpp synthetase	LL195	combined	1.53	4.41E-02
BN389_22610	PBP4 class A penicillin-binding protein	LL195	combined	1.49	3.00E-02
BN389_06450	Uncharacterized ABC transporter ATP-binding protein TM_0288	LL195	combined	1.27	3.00E-02
*L. monocytogenes* EGD-e					
lmo2487	Hypothetical protein	EGD-e	10	6.94	1.38E-49
lmo2486	Hypothetical protein	EGD-e	10	6.64	6.53E-46
lmo2485	Hypothetical protein	EGD-e	10	6.08	1.68E-16
lmo2487	Hypothetical protein	EGD-e	3	5.17	1.94E-23
lmo2003	GntR family transcriptional regulator	EGD-e	10	5.13	1.25E-40
lmo2004	GntR family transcriptional regulator	EGD-e	10	4.79	2.52E-24
lmo2484	Hypothetical protein	EGD-e	10	4.3	1.64E-07
lmo2004	GntR family transcriptional regulator	EGD-e	3	4,13	5,49E-10
lmo2003	GntR family transcriptional regulator	EGD-e	3	4,11	1,74E-07
lmo2125	Sugar ABC transporter substrate-binding protein	EGD-e	10	4,04	2,13E-05
lmo2486	Hypothetical protein	EGD-e	3	4,03	6,76E-18
lmo0278	Sugar ABC transporter ATP-binding protein	EGD-e	10	3,54	6,21E-21
lmo2745	ABC transporter ATP-binding protein	EGD-e	10	3,44	1,37E-22
lmo0292	Heat-shock protein htrA serine protease	EGD-e	10	3.41	1.22E-14
lmo2485	Hypothetical protein	EGD-e	3	3.24	1.66E-02
lmo2001	PTS mannose transporter subunit IIC	EGD-e	10	3.23	9.13E-14
lmo2124	Sugar ABC transporter permease	EGD-e	10	2.93	3.70E-05
lmo0278	Sugar ABC transporter ATP-binding protein	EGD-e	3	2.53	8.78E-07
lmo2125	Sugar ABC transporter substrate-binding protein	EGD-e	3	2.52	3.08E-02
lmo2229	PBP4 class A penicillin-binding protein	EGD-e	10	2.46	9.34E-11
lmo1747	virB ABC transporter ATP-binding protein	EGD-e	10	2.42	5.53E-10
lmo1747	virB ABC transporter ATP-binding protein	EGD-e	3	2.32	1.06E-06
lmo2123	Sugar ABC transporter permease	EGD-e	10	2.32	3.00E-02
lmo2001	PTS mannose transporter subunit IIC	EGD-e	3	2.17	1.00E-04
lmo0802	(p)ppGpp synthetase	EGD-e	10	2.05	9.17E-05
lmo0607	ABC transporter ATP-binding protein	EGD-e	10	1.97	2.69E-06
lmo1020	liaF	EGD-e	10	1.88	2.52E-06
lmo1021	liaS	EGD-e	10	1.88	7.25E-08
lmo0292	Heat-shock protein htrA serine protease	EGD-e	3	1.73	3.85E-06
lmo1746	virA ABC transporter permease	EGD-e	10	1.5	9.88E-05
lmo1438	Penicillin-binding protein, PBP3	EGD-e	10	1.22	5.00E-03
lmo1746	virA ABC transporter permease	EGD-e	3	1.22	4.00E-04
lmo2555	lafA N-acetylglucosaminyl-phosphatidylinositol biosynthesis protein	EGD-e	10	1,2	4,37E-03
lmo2745	ABC transporter ATP-binding protein	EGD-e	3	1,14	2,64E-03
lmo1022	Two-component response regulator, liaR	EGD-e	10	1,1	1,34E-02
lmo2229	PBP4 class A penicillin-binding protein	EGD-e	3	1,06	7,11E-03
lmo0608	ABC transporter ATP-binding protein	EGD-e	10	1,02	2,00E-02
lmo1088	tagB (teichoic acid biosynthesis protein B)	EGD-e	3	−1,04	4,80E-02
lmo1088	tagB (teichoic acid biosynthesis protein B)	EGD-e	10	−1,23	4.40E-02
lmo0974	dltA (D-alanine--poly(phosphoribitol) ligase subunit 1)	EGD-e	10	−2,11	7.73E-05
*L. monocytogenes* 10403S					
LMRG_RS12625	Hypothetical protein	10403S	10	8.48	6.32E-57
LMRG_RS12620	Hypothetical protein	10403S	10	7.18	7.52E-39
LMRG_RS12625	Hypothetical protein	10403S	3	6.02	1.38E-18
LMRG_RS12610	Hypothetical protein	10403S	10	5.66	6.39E-07
LMRG_RS12620	Hypothetical protein	10403S	3	4.5	4.92E-13
LMRG_RS01515	Serine protease htrA	10403S	10	3.8	3.26E-21
LMRG_RS01420	Sugar ABC transporter ATP-binding protein	10403S	10	3.7	2.14E-19
LMRG_RS13990	ABC transporter	10403S	10	3.52	1.58E-14
LMRG_RS11825	ABC transporter ATP-binding protein	10403S	3	3.38	1.81E-02
LMRG_RS08825	virB ABC transporter, ATP-binding protein	10403S	10	2.81	5.15E-12
LMRG_RS04025	(p)ppGpp synthetase	10403S	10	2.8	1.11E-09
LMRG_RS08820	virA ABC-type antimicrobial peptide transport system, permease component	10403S	10	2.42	1.91E-05
LMRG_RS03040	ABC transporter, ATP-binding protein	10403S	10	1.96	6.56E-06
LMRG_RS04025	(p)ppGpp synthetase	10403S	3	1.84	4.78E-02
LMRG_RS01515	Serine protease htrA	10403S	3	1.83	6.48E-06
LMRG_RS11305	PBP3 penicillin-binding protein	10403S	10	1.79	3.36E-05
LMRG_RS05140	liaF	10403S	10	1.76	4.74E-05
LMRG_RS07155	Penicillin-binding protein	10403S	10	1.65	2.74E-05
LMRG_RS05145	liaS	10403S	10	1.63	1.80E-04
LMRG_RS03045	ABC transporter	10403S	10	1.62	7.13E-05
LMRG_RS13990	ABC transporter	10403S	3	1.60	2.18E-02
LMRG_RS05150	liaR	10403S	10	1.51	4.02E-03
LMRG_RS12965	lafA 1,2-diacylglycerol 3-glucosyltransferase	10403S	10	1.43	1.38E-03
LMRG_RS03040	ABC transporter, ATP-binding protein	10403S	3	1.24	3.77E-02
LMRG_RS12795	tagA	10403S	Combined	−1,37	4.81E-02
LMRG_RS04420	ydbT	10403S	10	−1,84	3.10E-02

Across all three *L. monocytogenes* strains tested (LL195, EGD-e, and 10403S), exposure to carrot juice led to a pronounced upregulation of multiple genes, whereas only a few loci were consistently downregulated. In both EGD-e and 10403S, the most strongly upregulated genes were predominantly annotated as hypothetical proteins, and in the EGD-e GntR family, transcriptional regulators were also among the top upregulated loci. In EGD-e and 10403S, in addition to hypothetical proteins, the most strongly upregulated genes included ABC transporter components, (p)ppGpp synthetase, the heat-shock protease HtrA, and penicillin-binding proteins, with PTS mannose transporters particularly affected in EGD-e and teichoic acid biosynthesis genes in 10403S. Only a few genes were downregulated in EGD-e and 10403S, mainly related to cell envelope modification, including *dltA* in EGD-e and *ydbT* in 10403S. In LL195, the most strongly upregulated genes included the multidrug resistance transporter bmrA, additional ABC transporter components, (p)ppGpp synthetase, the heat-shock protease HtrA, and penicillin-binding proteins (Table 3).

### Genes involved in cell wall integrity, two-component systems, and ABC-transporters were significantly enriched in functional enrichment analysis

KEGG analysis was performed to identify the pathways that were affected in response to stress induced by carrot juice. Tables S4 and S5 show the full results yielded by the KEGG analysis. The most important pathways are shown in Table 4 and discussed below.

**TABLE 4:** Pathways affected in response to stress induced by carrot juice (for the full data set, refer to Tables S4 and S5)^*a*^

	*L. monocytogenes* strains and time points					
Pathway	EGD-e (3 min)	EGD-e (10 min)	10403S (3 min)	10403S (10 min)	LL195 (3 min)	LL195 (10 min)
ABC transporters	**↑**	**↑**	**↑**	**↑**	**↑**	**↑**
Beta-lactam resistance	**↑**	**↑**	**↑**	**↑**	**↑**	**↑**
Two-component systems	**↑**	**↑**	**↑**	**↑**	**↑**	**↑**
Amino acid biosynthesis	**↑**	**↑**	**↑**	**↑**	**↑**	**↑**
Biosynthesis of cofactors	**↑**	**↑**	**↑**	**↑**	**↑**	**↑**
Cationic antimicrobial peptide (CAMP) resistance	**↑**	**↑**	**↑**	**↑**	**–**	**↑**
Quorum sensing	**↑**	**↑**	**–**	**↑**	**↑**	**↑**
2-Oxycarboxylic acid metabolism	**–**	**↑**	**–**	**↑**	**–**	**↑**
Alanine, aspartate, and glutamate metabolism	**–**	**↑**	**–**	**↑**	**–**	**↑**
Fructose and mannose metabolism	**↑**	**↑**	**–**	**–**	**–**	**–**
Phosphotransferase system	**↑**	**↑**	**–**	**↑**	**–**	**–**
Glycolysis and gluconeogenesis	**↑**	**↑**	**–**	**↑**	**–**	**–**
Starch and sucrose metabolism	**↑**	**↑**	**–**	**↑**	**–**	**–**
Cysteine and methionine metabolism	**–**	**–**	**–**	**–**	**–**	**↑**
Nitrogen metabolism	**–**	**–**	**–**	**–**	**–**	**↑**
Sulfur metabolism	**–**	**–**	**–**	**–**	**–**	**↑**
Biosynthesis of secondary metabolites	**↓**	**↓**	**–**	**↓**	**↓**	**↓**
Two–component systems	**↓**	**↓**	**–**	**↓**	**↓**	**↓**
Amino acid biosynthesis	**–**	**↓**	**–**	**↓**	**–**	**↓**
ABC Transporters	**–**	**↓**	**–**	**↓**	**–**	**↓**
Alanine, aspartate, and glutamate metabolism	**↓**	**↓**	**–**	**–**	**↓**	**↓**
Bacterial chemotaxis	**↓**	**↓**	**–**	**↓**	**–**	**–**
Biosynthesis of cofactors	**↓**	**↓**	**–**	**↓**	**–**	**–**
Pyrimidine metabolism	**↓**	**↓**	**–**	**–**	**–**	**–**
Flagellar assembly	**–**	**↓**	**–**	**–**	**–**	**–**

^*a*^ (↑) indicates upregulation, (↓) indicates downregulation, (–) indicates no change.

Some KEGG pathways were significantly upregulated across all three strains and at both time points: genes involved in ABC transporters, the beta-lactam resistance pathway, two-component systems, amino acid biosynthesis pathways, and the biosynthesis of cofactors (full data in Table S4). Biosynthesis of cofactors was also significantly upregulated in response to carrot juice across all three strains, with the highest number of genes mapping to this pathway in EGD-e.

The data revealed time-dependent expression patterns: genes involved in 2-oxocarboxylic acid metabolism and also in alanine, aspartate, and glutamate metabolism were upregulated in all three strains after 10 min of exposure to carrot juice, but not after 3 min. Similarly, the number of genes associated with ABC transporters increased after 10 min compared with 3 min (Table 4).

Genes associated with cationic antimicrobial peptide (CAMP) resistance and quorum sensing were upregulated across all strains and showed late expression patterns in LL195 and 10403S, respectively.

**Fig 3:**
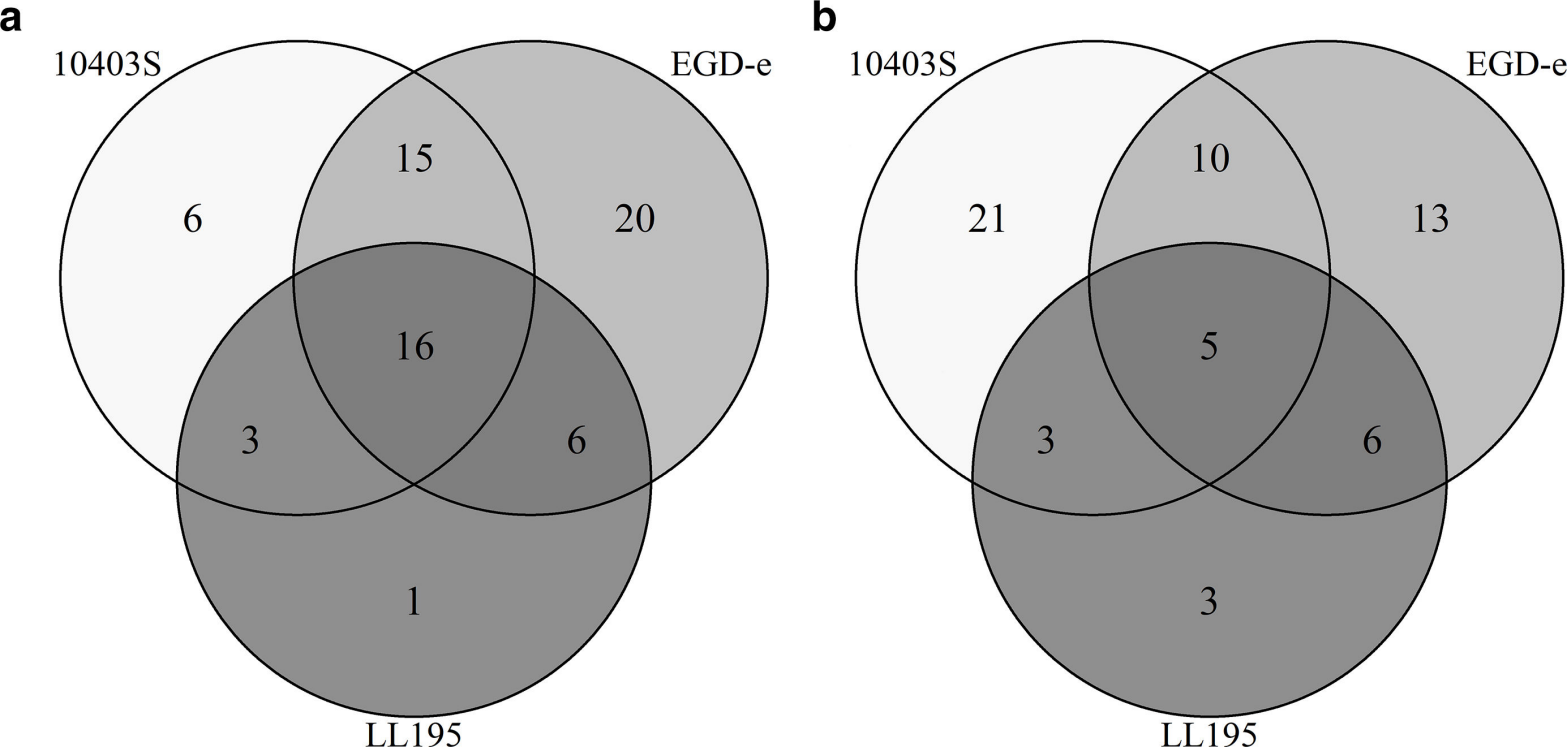
Venn diagrams of pathways upregulated (**a**) and downregulated (**b**) in all strains of *L. monocytogenes* and common ones between the strains. Pathways were derived from significantly differentially expressed genes (DEGs).

Furthermore, we observed pathways unique to individual strains (Fig. 3), some also showing time-dependent expression patterns. Twelve genes that were involved in fructose and mannose metabolism in EGD-e were upregulated after 10 min of exposure, and five of these genes were also upregulated after 3 min as an early response mechanism. Genes with functions in phosphotransferase systems were predominantly upregulated in EGD at both the 3 min and 10 min time points, with a noticeable increase in the number of involved genes over time. Two genes in the phosphotransferase systems (*LMRG_RS00135* and *LMRG_RS13510*) were also upregulated in 10403S exclusively at the 10-min time point. The glycolysis and gluconeogenesis, starch, and sucrose metabolism pathways were found enriched mostly in EGD-e, with two genes mapping to either of these in 10403S as well.

In LL195, cysteine and methionine metabolism, nitrogen metabolism, and sulfur metabolism showed a time-dependent upregulation after 10 min (Table 4).

Genes involved in the biosynthesis of secondary metabolites and two-component systems were downregulated across all strains (Table 4). This was observed at both time points in LL195 and EGD-e, and after 10 min in 10403S. Although many genes involved in the biosynthesis of amino acids and ABC transporters were upregulated, some of them were also downregulated across all three strains after 10 min of exposure.

Genes related to alanine, aspartate, and glutamate metabolism were downregulated in EGD-e and LL195 at all time points but not in 10403S. Bacterial chemotaxis and biosynthesis of cofactors were downregulated in EGD-e and 10403S, with a late response in 10403S only after 10 min. Furthermore, no pathways were downregulated in 10403S after 3 min post-exposure (Table 4).

In EGDe, pyrimidine metabolism was downregulated at both time points, whereas flagellar assembly and ribosome-related genes were downregulated only after 10 min (Table 4).

## DISCUSSION

### Screening of 52 carrot accessions for their antilisterial effect

An important question addressed in this study was whether the observed antilisterial effect is a stable characteristic of individual carrot accessions and whether it persists over multiple harvest years. Starting with 52 accessions, we selected the three with the strongest effect and confirmed their antilisterial activity against *L. monocytogenes* LL195 over three harvest periods. This demonstrates that the specific type of carrot crucially determines its antilisterial properties, a finding consistent with previous work showing that six different types of carrots sourced from supermarkets exhibited significantly different levels of antilisterial activity (31).

Although the effect persisted throughout the harvesting periods, particularly in the carrots exhibiting the strongest effect in this study, its intensity varied substantially between these periods. One possible explanation is that the screening in the second year was conducted using a higher inoculum (7-log_10_ vs 4-log_10_ CFU/mL), which was done to discriminate more effectively among carrots that had displayed antimicrobial activity in the first harvesting period. These differences in effect could furthermore be attributed to variations in environmental factors influencing carrot cultivation, for example, weather conditions, soil composition, or nutrient availability, etc. (32). Moreover, the profile of phenolic compounds in carrot cultivars has been reported to vary across harvesting periods, even when grown at the same location (33). This is relevant because phenolic phytoalexins, such as 6-methoxymellein, have been proposed as candidate compounds with antimicrobial activity (25). It has also been established that carbohydrates in carrots were influenced by pre-harvesting factors such as temperature and the nature of the soil (34). Additionally, during storage, there can be considerable environmental fluctuations across harvesting periods in phenolic compounds (35), as well as in polyacetylenes like falcarinol and falcarindiol, laserin, anthocyanin, carotene, and lycopene (TN, personal communication, unpublished data). Taken together, these observations suggest that the variability in the effect size across harvest periods likely reflects both the different inoculum levels of *L. monocytogenes* LL195 and fluctuations in carrot chemical composition, which may in turn influence the abundance of compounds responsible for their antilisterial properties.

*L. monocytogenes* LL195 was more resistant to carrot juice than the two other strains. This confirms our hypothesis that the effect is strain-specific. Although strain LL195 is a human outbreak serotype 4b strain, EGD-e, and 10403S are 1/2a laboratory strains. The lower resilience of EGD-e and 10403S may be partly explained by the fact that these laboratory strains have lost some of their natural stress resilience during multiple passages in laboratory media (36). For example, EGD-e also showed different behavior compared with other strains under acid (37) and salt stress (38) and was less invasive in eukaryotic host cells (38). *L. monocytogenes* 10403S was less heat-tolerant compared with 14 other strains (39). More research is required to explore the variations observed between strains.

### Transcriptome changes in *L. monocytogenes* strains following exposure to carrot juices

To investigate the transcriptomic changes in three *L. monocytogenes* strains in response to carrot juice after 3 and 10 min, each strain was individually inoculated into freshly made carrot juice, using three carrots with a strong antilisterial effect and three with no observable antilisterial effect.

#### Structure and integrity of the cell wall

Many genes that are functionally associated with the structure and integrity of the cell wall were upregulated in response to exposure to carrot juice, suggesting that the cell wall is the primary target of the active compound in carrots. Among them were penicillin-binding proteins, the cell wall stress response kinase LiaS, lipoteichoic acid biosynthesis genes, and genes involved in resistance against cationic antimicrobial peptides (CAMPs) that act on the cell wall.

A class B penicillin-binding protein (40), PBP3, was significantly upregulated in 10403S (*LMRG_RS07155*, 1.65 log_2_FC) and EGD-e (*lmo1438,* 1.23 log_2_FC) after 10 min (Table 3) of exposure to carrot juice. Class B PBPs lack a transglycosylase domain and instead contain a noncatalytic domain, which is thought to play a role in cell morphogenesis by interacting with other proteins involved in the cell cycle (40, 41). Others found that the disruption of *lmo1438* in EGD-e resulted in increased susceptibility to all tested β-lactams such as ampicillin, amoxicillin, and gentamicin (41, 42). In all three *L. monocytogenes* strains, a class A penicillin-binding protein 4 (PBP4) was upregulated, *lmo2229* (1.05 log_2_FC after 3 min, 2.46 after 10 min) in EGD-e at both time points and *BN389_22610* (1.49 log_2_FC) in LL195 in the combined data set, and *LMRG_RS11305* (1.79 log_2_FC) in 10403S only after 10 min. As a multimodular class A protein, PBP4 is the primary bifunctional peptidoglycan synthase, possessing both glycosyltransferase (GT) and transpeptidase (TP) activities (43, 44). Others have shown that overexpression of PBP3 leads to elevated levels of PBP4, indicating a tightly regulated interaction between these PBPs to maintain cell wall integrity and facilitate cell division (45).

Genes encoding the three-component system LiaFSR (46), including the cell-wall stress response kinase liaS (46), were upregulated in response to carrot-induced stress in *L. monocytogenes* EGD-e and *L. monocytogenes* 10403S (in EGD-e 1.88 log_2_FC after 10 min, and in 10403S 1.63 after 10 min). Further support for the cell wall as the primary target of the active compound in carots comes from the overlap of differentially expressed genes in this study with the response to nisin, an antibacterial peptide that interacts with the peptidoglycan precursor lipid II and causes pore formation in the cytoplasmic membrane (47): the LiaFSR system in *L. monocytogenes* enhances resistance to nisin (48) and is activated in response to cell wall-active antimicrobials (46, 49, 50). Another study showed that the upregulation of PBP4, *lmo2487,* and liaS in *L. monocytogenes* 412 led to increased resistance to nisin (47) - *lmo2487* was the gene with the highest fold change in EGDe in our study, with a 5.17 log_2_FC after 3 min and 6.94 after 10 min. In a study aimed at characterizing the liaFSR three-component system, a total of 27 genes were differentially expressed in an *L. monocytogenes* mutant lacking the histidine kinase LiaS (46). A subset of these genes was also upregulated in our study: the ABC transporter ATP Binding Protein, PBP4, LiaI, and LiaH, and some hypothetical proteins (46), supporting a role of LiaFSR in response to carrot juice. Among the genes most strongly regulated by this system were *lmo2484 to lmo2487*, among which *lmo2484* and *lmo2485* have also been implicated in nisin resistance (51, 52).

The *lafA* (lipoteichoic acid [LTA] anchor formation protein A) gene that encodes for an N-acetylglucosaminyl-phosphatidylinositol biosynthesis protein (53) was upregulated in EGD-e (*lmo2555*, 1.22 log_2_FC) and 10403S (*LMRG_RS12965*, 1.43 log_2_FC) after 10 min. This gene is required for the formation of glucosyl-diacylglycerol (Glc-DAG), a glycolipid that anchors the LTA polymer to the bacterial membrane in *L. monocytogenes* (52, 53). The deletion of *lafA* also led to increased susceptibility to nisin (52). Its upregulation in carrot juice suggests that *lafA* contributes to resistance to various cell envelope-targeting antimicrobials.

In *L. monocytogenes* 10403S and EGD-e, among the most upregulated genes in response to carrot-induced stress are genes that encode hypothetical proteins (from *LMRG_RS12610-LMRG_RS12625* in 10403S ranging from 3,4 to 6 log_2_FC after 3 min and from 5,66 to 8,48 after 10 min, corresponding to *lmo2484-lmo2487* in EGD-e, ranging from 3,24 to 5,17 log_2_FC after 3 min and from 4,3 to 6,94 after 10 min). These hypothetical proteins contain domains that have been associated with cell wall stress response in *Bacillus subtilis* (54, 55). One of these proteins, *LMRG_RS12625,* corresponding to *lmo2487,* is similar to LiaX (lipid-II-interacting antibiotics X), which is involved in the resistance to antimicrobial peptides in *Enterococcus faecalis* (56–58) and activates the cell membrane stress response in *L. monocytogenes* (59). *LMRG_RS12620*, corresponding to *lmo2486*, encodes a PspC (phage shock protein C) domain-containing transcriptional regulator and plays a critical role in sensing membrane stress and restoring envelope integrity in *Bacillus* (55). *L. monocytogenes* does not possess a PSP system in the strict sense, and cell envelope stress response is mediated by two-component systems such as LisRK or CesRK (49, 60–62). The presence of Psp-related domains among the genes upregulated in *L. monocytogenes* strains 10403S and EGD-e after carrot-induced stress suggests that these strains may employ an alternative but functionally analogous system to manage membrane stress, potentially relying on protein–protein interactions and domain-mediated signaling to preserve membrane integrity and promote survival under adverse conditions.

The KEGG analysis highlighted the cationic antimicrobial peptide (CAMP) resistance pathway as significantly overrepresented among the upregulated genes. CAMPs are naturally occurring broad-spectrum antibiotics that act on the inner membrane of bacteria and are produced by nearly all life forms to combat infections (63). It is plausible that the proteins expressed in response to cell wall damage caused by CAMPs also serve a function in response to other cell wall-targeting agents, leading to their upregulation under carrot stress.

On the other hand, genes that are involved in teichoic acid biosynthesis were downregulated in response to carrot stress: *tagB* in EGD-e (*lmo1088*, −1,04 log_2_FC after 3 min, −1,23 after 10 min), *tagA* in 10403S (*LMRG_RS12795*, −1,37 log_2_FC in the combined data set), *dltA* (*lmo0974*, −2,11 log_2_FC after 10 min) in EGD-e, and *ydbt* in 10403S (*LMRG_RS04420*, −1,84 log_2_FC after 10 min). These genes are known to contribute to antimicrobial resistance in gram-positive bacteria. For instance, *ydbT* has been implicated in the resistance against antimicrobials in *Staphylococcus aureus* (64). Similarly, deleting the *dltA* gene in *Lactococcus garvieae* increased resistance to nisin (65). The dlt operon was shown to be the key contributor to resistance against antimicrobial compounds in *L. monocytogenes* (66)*,* which may resist such stress by altering surface charge through changes in teichoic acids and membrane lipids (67). This adaptive strategy may also involve modifying the cell wall core structure (68). The upregulation of genes associated with cell wall biosynthesis in all three strains suggests once more that carrot exposure imposes significant stress on the *L. monocytogenes* cell wall that triggers a complex bacterial response to maintain cell wall integrity.

#### Two-component systems and ABC transporters

Although a response to maintain cell wall integrity seems to be the major transcriptional change in *L. monocytogenes* after exposure to carrot juice, other responses might play a supporting role. Multiple genes associated with regulatory systems were significantly upregulated across all strains and time points. One of these genes, *htrA*, a heat-shock protein serine protease, was significantly upregulated in both *L. monocytogenes* EGD-e (*lmo0292*, 1.73 log_2_FC after 3 min, 3.41 after 10 min) and 10403S (*LMRG_RS01515*, 1.83 log_2_FC after 3 min, 3.8 after 10 min). *HtrA* plays a role in stress responses such as heat, cell wall antibiotics, and acid stress, not only in *Listeria* but also across diverse bacterial species, including both gram-positive and gram-negative organisms (69–73). The three-component system LiaFSR (upregulated in *L. monocytogenes* EGD-e and *L. monocytogenes* 10403S after carrot-induced stress) was not only involved in cell wall-associated stress but also in the response to heat, salt, and pH stress (74).

The ABC transporters, which serve as efflux pumps in *L. monocytogenes*, help the bacteria resist toxic compounds, including disinfectants, antibiotics, and antimicrobial peptides such as nisin (75–77). Many of the upregulated genes identified in our study encode ABC transporter ATP-binding proteins: *LMRG_RS13990* (3.52 log_2_FC after 10 min), *LMRG_RS03040* (1.24 log_2_FC after 3 min, 1.96 after 10 min), and *LMRG_RS03045* (1.62 log_2_FC after 10 min) in 10403S; *lmo2745* (1.14 log_2_FC after 3 min, 3.44 after 10 min), *lmo0607* (1.97 log_2_FC after 10 min), and *lmo0608* (1.02 log_2_FC after 10 min) in EGD-e; and *BN389_27200* (3.36 log_2_FC after 3 min, 4.19 after 10 min), *BN389_06440* (1.56 log_2_FC in the combined data set), and *BN389_06450* (1.27 log_2_FC in the combined data set) in LL195; an amino acid ABC transporter ATP-binding protein *LMRG_RS11825* (3.38 log_2_FC after 3 min) in 10403S; and ABC transporters related to sugar uptake *LMRG_RS01420* (3.7 log_2_FC after 10 min) in 10403S, *lmo0278* (2.53 log_2_FC after 3 min, 3.54 after 10 min), *lmo2125* (2.52 log_2_FC after 3 min, 4.04 after 10 min), *lmo2124* (2.93 log_2_F after 10 min), and *lmo2123* (2.32 log_2_FC after 10 min) in EGD-e. One transporter, *lmo2745*, is under the control of LiaSR and shows high similarity to a multidrug resistance ABC transporter responsible for exporting toxic compounds in *B. subtilis* (78).

The genes *virA* (*lmo1746,* 1.22 log_2_FC after 3 min, 1.5 after 10 min*, LMRG_*RS08820, 2.42 log_2_FC after 10 min) and *virB* (*lmo1747,* 2.32 log_2_FC after 3 min, 2.42 after 10 min*, LMRG_RS08825,* 2.81 log_2_FC after 10 min)*,* upregulated in both EGD-e and 10403S, encode components of an ABC transporter. These proteins are essential for the function of VirR, the response regulator of the VirRS two-component system in *L. monocytogenes* (79). VirAB-regulated VirR is involved in virulence, cell wall integrity, and defense against cell envelope stress caused by antimicrobial peptides such as nisin, as well as antibiotics like cefotaxime and bacitracin, and disinfectants such as benzalkonium chloride (66, 75, 79–83). Hence, the VirAB and VirR regulation provides another link between carrot juice and cell wall integrity.

#### PTS systems

Mainly in *L. monocytogenes* EGD-e, our data showed upregulation of various phosphotransferase systems involved in sugar transport after exposure to carrot juice, for example, *lmo2001* (2,17 log_2_FC after 3 min, 3,23 after 10 min). In bacteria, PTS systems are known to play regulatory roles, particularly in Firmicutes, where they regulate transcription factors and both PTS-related and non-PTS permeases. Additionally, these systems also regulate physiological processes such as biofilm formation, virulence (84, 85), and environmental stress responses, such as acid, osmotic, cold, and heat stresses, in many bacteria, including *L. monocytogenes* (86–94). Taken together, our results suggest that PTS systems may play a crucial role in response to the stress that is caused by carrots on the cell wall, either through their function in carbohydrate metabolism or through secondary regulatory functions.

#### Purine metabolism

The (p)ppGpp synthetase was upregulated in all three *L. monocytogenes* strains following carrot stress: *lmo0802* (2.05 log_2_FC after 10 min) in EGD-e, *LMRG_RS04025* (1.84 log_2_FC after 3 min, 2.8 after 10 min) in 10403S, and *ywaC* (1.53 log_2_FC in the combined data set) in LL195. (p)ppGpp is a key component of the stringent response in bacteria, which adaptively regulates metabolism under stress conditions, such as amino acid deficiency, by prioritizing amino acid synthesis over ribosome production (95). However, at this stage, it is unclear whether carrot stress leads to amino acid deficiency. Notably, *relA*, the central regulator of the stringent response, was not differentially regulated in any of the three *L. monocytogenes* strains. This suggests that the (p)ppGpp, which is upregulated across all strains, may enhance fitness in response to carrot stress through one of its global reprogramming functions.

In summary, this study reveals the transcriptomic responses of *L. monocytogenes* and shows that exposure to carrot juice evoked significant changes in the expression of genes mainly related to cell wall structure and integrity. In addition, two-component regulatory systems and ABC transporters were upregulated, reflecting the multifaceted response of *L. monocytogenes* against the carrot juice. In our study, a remarkable number of the regulated genes have previously been associated with resistance to nisin stress (48, 51, 52, 96, 97). Additionally, we found that certain downregulated genes were linked to decreased resistance to nisin stress (52, 65). This suggests that *L. monocytogenes* may employ a mechanism similar to that used for nisin resistance when responding to carrot-induced stress.

Collectively, these results reveal that carrot-induced stress triggers a robust and targeted bacterial response, emphasizing the potential of natural plant-derived compounds as antimicrobial agents. Understanding these responses at the molecular level not only advances our knowledge of bacterial stress adaptation but also provides valuable insights into the development of novel strategies to control *L. monocytogenes* in food products. Future studies should focus on identifying specific carrot-derived compounds responsible for these effects and exploring their potential applications in food safety and preservation.

## MATERIALS AND METHODS

### Bacterial strains and culture conditions

*L. monocytogenes* LL195, a serotype 4b strain from an outbreak in Switzerland lasting from 1983 to 1987 (98) (accession number HF558398.1), and two serotype 1/2 a strains, *L. monocytogenes* 10403S (accession number NC_017544.1) and *L. monocytogenes* EGD-e (accession number NC_003210.1), were used in this study. To create the inocula used in this study, the strains were individually streaked on Tryptic Soy Agar (by Merck KGaA, cat. No. 1054580500) from −80°C stocks and incubated for 24 h at 37°C. A few colonies were then precultured in 5 mL Tryptic Soy Broth (TSB; Merck KGaA, cat. No. 1054590500) and incubated at 37°C with agitation at 200 rpm overnight (minimum of 16 h). These pre-cultures were subcultured 1:100 into fresh TSB and grown until the strains reached the early exponential phase, corresponding to an optical density (OD_600_) of 0.4 and approximately 8-log_10_ CFU/mL. These cultures were then diluted in TSB, where necessary, to reach the required CFU/mL to be used in the experiments.

For quantitative analysis, *L. monocytogenes* LL195 and *L. monocytogenes* EGD-e were plated on Listeria Chromogenic Agar according to Ottaviani and Agosti (ALOA, by Merck KGaA, cat. no. MC1004270500. *L. monocytogenes* 10403S was plated on Oxford agar, as it did not grow efficiently on ALOA agar (by Merck KGaA, cat. No. 1.07004.0500, Supplement cat. No. 1.07006).

### Carrot strains and storage conditions

All carrot plants were grown under field conditions on the experimental field of the Julius Kühn Institute (JKI) in Quedlinburg (51° 470′ N, 11° 80′ E, North Harz Foreland, Loess-Black Earth (Lö 1 a), altitude: 140 m above sea level).

The seeds were sown in the first week of May, and the roots were harvested in mid-September. Additional irrigation was done as needed. After harvest, the carrots were manually cleaned of visible soil and stored at 4°C in plastic boxes with commercial sand to prevent them from spoiling.

In September 2021, we received 52 different carrot accessions, including commercial varieties, old landraces, breeding lines, and wild relatives. Some of the material has already been characterized in a previous study with regard to its genetic and chemical composition (99). They were selected for the presented experiments based on their different concentrations of several potential antimicrobial substances, such as 6-methoxymellein, falcarinol, falcarindiol, and laserines.

### Preliminary screening of the carrots for their antilisterial effect

To screen the effect of the 52 different carrot accessions on *L. monocytogenes* LL195, we used cold-pressed carrot juice. Cold-pressed juices were used to preserve bioactive compounds and maintain the natural chemical complexity of the juice, which may contribute to its antilisterial activity. Carrot juice was prepared as follows: whole carrots were rinsed under cold tap water, and approximately 1 cm from both ends of the carrots was cut off and discarded. The remaining carrot was then cut into disks and juiced with a commercial juicer (Panasonic MJ-L 500 Slow Juicer). The prepared carrot juices were then kept at room temperature and used within 30 min after preparation. In total, 990 µL of each batch of carrot juice was separately inoculated with 10 µL of *L*. *monocytogenes* LL195 to reach 4-log_10_ CFU/mL for the preliminary screening. The inoculated carrot juices were incubated at 4°C and sampled after 3, 15, 30, and 120 min. The samples from each time point were plated on the respective selective agar. An initial inoculation concentration of 4-log_10_ CFU/mL was selected as a starting point to enable observation of both an increase and a decrease in CFU/mL over several log units. For this, ALOA agar was used, and growth was assessed after incubation at 37°C for 48 h. The detection limit for the screening was 10 CFU/mL. All carrots were furthermore tested for the presence of naturally occurring *L. monocytogenes* with negative results (data not shown).

### Second screening of a subset of carrot accessions

The 19 accessions with the highest antilisterial potential in the first year were re-cultivated, harvested, and tested again the following year to verify the first screening results. The carrot juices were inoculated at a concentration of 7-log_10_ CFU/mL *L. monocytogenes* LL195, to allow the identification of effects over 4-log_10_ reductions. From these, the three strongest and three weakest carrot accessions were selected. Only these selected carrot accessions were grown for the next harvest. The screening protocol was repeated whenever newly harvested carrot roots were received to verify previous results.

The six selected carrot accessions were tested against *L. monocytogenes* strains EGD-e and 10403S and quantified on ALOA and Oxford agars, respectively, to verify the effect on these strains.

### Preparing and quantifying cultures for transcriptome analyses

During the initial screening of carrot accessions, we observed a rapid bactericidal effect of carrot juice on *L. monocytogenes*, which was not suitable for transcriptomic analysis. To enable RNA-seq, we therefore diluted the carrot juice to a concentration that produced approximately a 1-log₁₀ CFU/mL reduction in viable bacteria after 15 min of exposure. Preliminary experiments indicated that a 1:25 dilution in PBS achieved this effect while also reducing the amount of carrot tissue in the samples and thereby facilitating RNA isolation. Hence, for transcriptome analysis, 100 µL containing 7-log₁₀ CFU of *L. monocytogenes* strains LL195, EGD-e, and 10403S were inoculated separately into 900 µL of carrot juice diluted 1:25 in phosphate-buffered saline (PBS; Roth, cat. no. 0890.2) and incubated at 4°C. Samples for RNA isolation were collected after 3 min to capture the immediate transcriptional response to carrot-induced stress, and 10 min, to monitor early adaptation processes. To quantify residual *L. monocytogenes*, an additional sample was collected after 15 min, serially diluted, plated on ALOA/Oxford agar, and incubated at 37°C for 48 h to determine colony counts and calculate log₁₀ CFU/mL reductions. Carrot accessions used in these experiments included the three with the strongest antilisterial activity (nos. 13, 44, and 47) and the three with no detectable effect (nos. 3, 30, and 31). Given the high reproducibility of RNA-seq data (100), we treated the different carrot accessions and *Listeria* strains as biological replicates of one another, without including technical replicates. Our rationale was that genes consistently identified as differentially expressed across these replicates are more likely to represent biologically meaningful responses.

### RNA isolation

Samples were centrifuged immediately at 4°C and 7,000 × *g* for 5 min, the supernatant was discarded, and RNA was isolated using the Qiagen RNeasy Power Fecal Pro Kit (cat. no. 78404), according to the manufacturer’s instructions. After RNA isolation, samples were treated with DNase (Invitrogen TURBO DNA-*free* Kit, cat. no. AM1907) to reduce genomic DNA contamination in the samples. DNase-treated samples were stored at −80°C until further analysis.

*L. monocytogenes* cultures grown in TSB were used as controls. These samples were treated identically to samples that were exposed to carrot juices. Two control conditions were included to ensure that observed gene regulation could be attributed specifically to the antilisterial effect of active carrots. The first control was each strain of *L. monocytogenes* inoculated into tryptic soy broth (TSB) and incubated at 4°C to account for changes in gene expression due to low-temperature incubation. In addition, TSB provided an optimal growth condition that served as a reference baseline for comparison with the stress imposed by carrot juice. The second control was *L. monocytogenes* inoculated into carrots lacking antimicrobial activity to distinguish transcriptional responses related to the transition into carrot juice as a growth medium from those triggered by the antimicrobial properties of active carrots.

### Quality control and cDNA conversion

The integrity and quality of the RNA samples were assessed using an Agilent Bioanalyzer 2100 (model number G2939BA) with the 6000 RNA Nano Kit (cat no. 5067-1511) and via PCR for controlling for residual genomic DNA, and then transcribed into cDNA (Supplementary Word).

### Transcriptome sequencing

cDNA library preparation and total RNA sequencing were performed by Novogene Co., Ltd. (Munich, Germany). Sequencing was conducted using the Illumina NovaSeq X Plus Sequencing system, with 150 bp paired-end reads, following standard procedures. The RNA-seq data generated in this study have been deposited in the NCBI Sequence Read Archive (SRA) under accession number PRJNA1338360.

Transcriptome data analysis was essentially performed as described previously (101), with the exception that Bowtie2 v2.4.5 (102) was used for read mapping instead of BWA. All analyses were done with standard settings. The RNA-seq reads were mapped against the respective *L. monocytogenes* genomes EGD-e, LL195 (98), and 10403S. Normalization and determination of significant differential expression were performed in R v4.4.1 (R-project.org) using the Generalized Linear Models method in the EdgeR package v4.2.1 as described previously (103). Genes with an FDR ≤ 0.05 and > 2-fold differential expression were considered significantly differentially expressed.

The transcriptomes of three *L. monocytogenes* strains (LL195, EGD-e, and 10403S) were analyzed after exposure to the three carrot accessions with the strongest effect and compared with those exposed to the control carrots with no effect or to culture medium (TSB). Gene expression levels were evaluated at two time points—3 min and 10 min. A separate analysis was conducted using data merged from both time points.

### Kyoto Encyclopedia of Genes and Genomes (KEGG) analysis

For pathway analysis using KEGG (104), an FDR threshold of 0.1 was used to identify a broader range of genes within the pathways. The analysis was performed using the combined data from the 3 min and 10 min post-exposure time points to identify the overarching pathways involved in the stress response. Additionally, KEGG analysis was conducted separately for the 3-min and the 10-min results to facilitate a comparison between the early and late responses.

## SUPPLEMENTAL MATERIAL

Supplemental text spectrum.02091-25-s0001.docx Description of PCR used in the protocol.

Table S1 spectrum.02091-25-s0002.xlsx All significantly differentially expressed genes in *L. monocytogenes* 10403S across all time points

Table S2 spectrum.02091-25-s0003.xlsx All significantly differentially expressed genes in *L. monocytogenes* EGD-e across all time points.

Table S3 spectrum.02091-25-s0004.xlsx All significantly differentially expressed genes in *L. monocytogenes* LL195 across all time points.

Table S4 spectrum.02091-25-s0005.xlsx All significantly upregulated KEGG Pathways identified for each strain and time point along with the upregulated genes associated with these pathways.

Table S5 spectrum.02091-25-s0006.xlsx All significantly downregulated KEGG Pathways identified for each strain and time point along with the downregulated genes associated with these pathways.

ASM does not own the copyrights to Supplemental Material that may be linked to, or accessed through, an article. The authors have granted ASM a non-exclusive, world-wide license to publish the Supplemental Material files. Please contact the corresponding author directly for reuse.
