# Supplementary material for: Transcriptome analysis suggests the cell wall as the primary target of the antilisterial effect of carrots
Source: Microbiol Spectr. 2025 Nov 25;14(1):e02091-25. doi: 10.1128/spectrum.02091-25 (PMC12772317; doi:10.1128/spectrum.02091-25)
Supplement: Supplemental text — Description of PCR used in the protocol. [file spectrum.02091-25-s0001.docx]

Genomic DNA was quantified based on *rpoB* (Forward Primer Sequence: 5’-TCGTCGTCTTCGTTCTGTTG-3’; Reverse Primer Sequence: 5-’GTTCGCCAAGTGGATTTGTT-3’), a housekeeping gene of *L. monocytogenes*.

The RT-qPCR mixture contained 1X of SensiFAST SYBR No-ROX Mix (Bioline, cat. No. BIO-98005-BL) and 0.4 µM of each Primer. The qPCR program was designed as follows: pre-denaturation at 95 °C for 5 min, followed by 40 cycles of denaturation at 95 °C for 10 sec, primer annealing at 58 °C for 30 sec, and elongation at 72 °C for 15 sec. The amount of residual genomic DNA (gDNA) contamination in the RNA samples was quantified using a standard curve generated from *L. monocytogenes* gDNA at known copy numbers (from 3x10^1^ to 3x10^5^) to enable accurate determination of gDNA levels in the samples.

RNA was then reverse transcribed, using the Invitrogen Superscript IV First Strand cDNA synthesis System (cat. Number 18031200) with random hexamers as primers, according to the manufacturer's protocol. RT-qPCR was employed as described above, to quantify the cDNA after the conversion step.
